# Factors Associated with the Need for Support Among Informal Caregivers During Prolonged Hospitalization

**DOI:** 10.3390/healthcare14152420

**Published:** 2026-08-06

**Authors:** Carolina Pérez-Corraliza, María Yolanda Castaño-Blanco, José Manuel Romero-Perea, Laura Muñoz-Bermejo

**Affiliations:** 1 Department of Nursing, University Center of Merida, University of Extremadura, 06800 Merida, Spain; carolinapc@unex.es; 2 Department of Nursing, Faculty of Medicine and Health Sciences, University of Extremadura,06006 Badajoz, Spain; 3 Department of Nursing, Don Benito-Villanueva Hospital, 06400 Don Benito, Spain; jose.romerop@salud-juntaex.es; 4 Social Impact and Innovation in Health (INHealth) Research Group, University Center of Merida, University of Extremadura, 06800 Merida, Spain

**Keywords:** informal caregivers, hospitalization, perceived social support, mental health, caregiver burden

## Abstract

**Background/Objectives**: Prolonged hospitalization affects not only patients but also informal caregivers, who often bear a greater physical and emotional burden. The aim of this study was to analyze the burden, emotional well-being, perceived social support and family functioning among informal caregivers of patients undergoing prolonged hospitalization, as well as to identify the factors associated with the need for support. **Methods**: A descriptive-correlational cross-sectional study was conducted involving 94 informal caregivers of patients who had been hospitalized for seven days or more. Sociodemographic and care-related variables were collected, along with measures of caregiver burden (Zarit Burden Interview), emotional well-being (GHQ-12), and perceived social (Duke-UNC-11) and family support (Family APGAR). Caregivers who reported needing support were compared with those who did not. Furthermore, a binary logistic regression analysis was performed to identify factors independently associated with the need for support. **Results**: Caregivers who reported needing support exhibited higher levels of caregiver burden (*p* = 0.034), poorer emotional well-being (*p* = 0.010), lower perceived social support (*p* = 0.014) and poorer family functioning (*p* < 0.001) than those who did not require support. Significant differences were also observed in the distance between home and the hospital (*p* = 0.041) and in the length of the hospital stay (*p* = 0.001). Logistic regression analysis showed that family functioning (OR = 0.749) and length of hospital stay (OR = 1.523) were independently associated with the need for support. **Conclusions**: Poorer family functioning and a longer hospital stay were associated with a greater need for support among caregivers. Early identification of these factors can help in the design of interventions aimed at improving caregivers’ well-being while their relatives are in hospital.

## 1. Introduction

Prolonged hospitalization, defined as a stay of more than seven days [1], is a significant clinical phenomenon among patients with chronic illnesses, multiple conditions, high levels of functional dependency, or complex conditions requiring intensive monitoring. Although research has traditionally focused on the patient, in recent decades there has been growing interest in understanding the impact that this type of hospitalization has on primary informal caregivers, defined as those individuals who take on the responsibility of providing ongoing care and support without monetary compensation [2].

According to the Spanish National Institute of Statistics, the most common profile of an informal caregiver is that of a woman aged between 45 and 64, usually the patient’s daughter or spouse, with a primary school education. These individuals often have to balance caregiving with other family or work responsibilities. Evidence shows that between 60% and 70% of caregivers of chronic patients experience some degree of overload, emotional fatigue or deterioration in physical health, effects that can intensify during the family member’s hospitalization [3]. Although hospital admission may temporarily provide relief from caregiving tasks, it also often causes stress arising from adapting to the hospital environment, coordinating with healthcare staff, and uncertainty about the patient’s clinical progress [4,5].

One of the most widely studied aspects is perceived burden, defined as the physical, emotional, social and financial strain associated with long-term care [6]. This can be assessed using validated tools such as the Zarit Burden Interview. Various studies have demonstrated its link to a poorer quality of life, increased risk of anxiety and depression, and reduced coping ability [4,7]. During hospitalization, a sense of loss of control and clinical uncertainty can exacerbate this burden [8].

Perceived social support is an important protective factor that influences a caregiver’s coping ability and well-being [2]. Tools such as the Duke-UNC-11 Functional Social Support Questionnaire (Duke-UNC-11) enable the assessment of the emotional and practical support provided by family members, friends or community networks. Adequate social support is associated with lower levels of burden and better emotional health, while its absence increases the risk of anxiety, depression and social isolation [9,10]. This aspect is particularly relevant in rural or semi-urban settings, where support resources may be more limited [11].

Furthermore, family functioning plays a key role in the caregiver’s experience. The Family APGAR can be used to assess the family unit’s capacity for adaptation, communication and the distribution of responsibilities. Functional families promote a more balanced distribution of caregiving tasks and provide emotional support, reducing the perception of burden and facilitating effective coping strategies [11,12]. Conversely, family dysfunction is associated with higher levels of stress and emotional distress.

The mental health of the caregivers is another key factor, as prolonged hospitalization can trigger or exacerbate symptoms of anxiety, depression and psychological exhaustion [13]. Goldberg General Health Questionnaire (GHQ-12) [14] helps to identify these issues and facilitates early intervention where necessary. Early identification of psychological care needs is essential to prevent the condition from becoming chronic and to preserve the caregivers’ ability to perform their role without compromising their own well-being [15].

The length of the hospital stay is also a significant factor. Although the first few days may be perceived as a period of relief due to the partial delegation of care to healthcare staff, prolonged stays are often associated with increased emotional fatigue, uncertainty and a sense of loss of control. Similarly, the patient’s level of dependency directly influences the intensity of the burden, as patients with greater functional needs require more active involvement from the carer even during hospitalization [16].

In Spain, and particularly in ageing and geographically dispersed autonomous communities such as Extremadura, the role of the informal caregiver takes on particular significance. The limited availability of social and healthcare resources, combined with close family ties, encourages caregivers to play an active role in the care process. However, there remain significant gaps in our understanding of the multidimensional impact of prolonged hospitalization on this group.

Previous research has consistently shown that informal caregivers of hospitalized patients experience substantial caregiver burden, psychological distress, and reduced quality of life, particularly when caregiving demands are prolonged or intensive [17,18,19]. However, the factors associated with caregivers’ perceived support needs during prolonged hospitalization remain poorly understood.

Systematic reviews have identified patients’ functional dependence, the intensity and duration of caregiving, and caregivers’ social and family resources as key factors associated with caregiver outcomes [18]. However, these factors have often been examined in isolation, and little is known about how they are jointly associated with caregivers’ perceived support needs during prolonged hospitalization.

To provide a theoretical framework for this study, we adopted Pearlin’s Stress Process Model [20]. This model proposes that caregivers’ outcomes arise from the interaction between contextual characteristics, caregiving demands, and the personal and social resources available to the caregiver. Accordingly, it offers a suitable framework for examining the factors independently associated with caregivers’ perceived support needs during prolonged hospitalization. Based on Pearlin’s Stress Process Model, the variables included in this study were selected to represent the model’s main domains. Caregivers’ sociodemographic characteristics of caregivers, patients’ functional dependence, the intensity of care and the length of hospitalization were considered contextual characteristics and primary stressors. Caregiver burden and psychological distress, assessed through symptoms of anxiety and depression, represented the psychological outcomes of the stress process. Perceived social support and family functioning were included as mediating resources that may buffer the negative effects of caregiving-related stress. Based on this theoretical framework, we hypothesized that these contextual, psychological, and family-related factors would be independently associated with caregivers’ perceived need for support during prolonged hospitalization.

Despite growing evidence regarding the importance of informal caregivers, most studies have focused on the home setting and have not analyzed variables such as the length of hospital stay, the patient’s level of dependency, perceived social support, family functioning and emotional well-being. It is therefore necessary to generate specific evidence in contexts such as Extremadura. The results will enable the development of intervention strategies aimed at reducing caregiver burden, improving the caregiver’s emotional well-being and optimizing the quality of care provided to the patient.

For all these reasons, the aim of this study was to examine the psycho-emotional, family, and caregiving-related factors associated with the perceived need for support among informal caregivers of patients experiencing prolonged hospitalization, and to identify predictors of the need for support in this population.

## 2. Materials and Methods

### 2.1. Design

A cross-sectional observational study with a descriptive-correlational design was carried out in three public hospitals in Extremadura, Spain (the Badajoz University Hospital, the Mérida Hospital and the Don Benito-Villanueva University Hospital). A non-probabilistic convenience sampling strategy was used to select participants. Data were collected at a single point in time to assess levels of caregiver burden, emotional well-being and perceived social support amongst informal caregivers of patients with prolonged hospital stays; to compare these variables between caregivers who expressed a need for support and those who did not; and to determine the predictive role of these psycho-emotional factors on the perceived need for support.

### 2.2. Ethics

This study has been reviewed by the Bioethics and Biosafety Committee of the University of Extremadura, under reference number 212/2026. The study has been designed in accordance with the principles set out in the Declaration of Helsinki (64th General Assembly of the World Medical Association, Fortaleza, Brazil, 2013) and in accordance with Law 14/2007 on Biomedical Research.

### 2.3. Participants

The sample consisted of 94 informal caregivers of patients undergoing prolonged hospitalization. Eligible caregivers were identified specifically in the Cardiology, Surgery and Urology departments of the participating hospitals and invited to take part by a researcher appointed by the research team.

To be eligible, participants had to meet the following inclusion criteria: (a) act as an informal caregiver for a person hospitalized for a period of seven days or more; (b) spend more than eight hours a day accompanying and supervising the patient, assisting with basic activities of daily living where necessary, and providing other forms of support related to the patient’s needs during hospitalization, and express their intention to continue providing such care during their relative’s hospitalization; and (c) have signed the informed consent form before the start of the study. The threshold of more than eight hours a day was established as an operational criterion to identify caregivers with a substantial and ongoing involvement in care, using the approximate duration of a standard eight-hour work shift as a reference. The time spent on care was assessed via self-report, with caregivers asked to estimate the approximate number of hours per day they spent providing care, supervision, companionship and support to the patient during their relative’s hospitalization.

Caregivers who had difficulties that prevented them from adequately understanding the questionnaires or completing the assessment tools independently were excluded.

A total of 115 potentially eligible caregivers were contacted. Of these, 14 declined to take part. Five participants were excluded: four did not meet the eligibility criteria relating to their involvement in care, specifically the requirement to devote more than eight hours a day to care tasks and to express their intention to continue providing care while their relative was hospitalized; and one participant had difficulties that prevented them from adequately understanding the questionnaires and completing the assessment tools independently. In addition, two questionnaires were incomplete.

**Figure 1:**
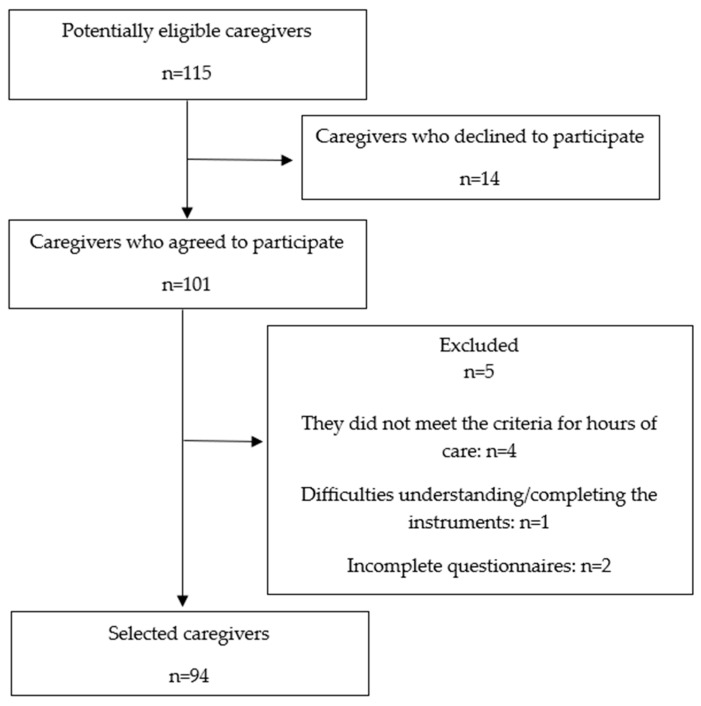
Flowchart of participant recruitment and selection.

Only one caregiver per patient was included, in order to ensure the independence of the observations. The flowchart (Figure 1) summarizes the process of identifying, assessing eligibility, recruiting and selecting participants.

### 2.4. Procedures and Measures

Data were collected in person from each caregiver using a standardized self-administered questionnaire in hospitals within the Autonomous Community of Extremadura (Spain). The administration of the questionnaires and the interviews were organized in advance by the research team and took place during March and April 2026.

The measures used are detailed below.

Outcome variable

The primary outcome was the perceived need for support in providing care during the relative’s hospitalization. This outcome was assessed using a single study-specific item: “Do you consider that you need support to provide care?” Responses were recorded as a dichotomous variable (“Yes” or “No”) and were self-completed by informal caregivers as part of the study questionnaire during the patient’s hospital stay. This item was specifically developed for the present study and was intended to assess caregivers’ perception of requiring additional support to meet the demands of caregiving in the hospital setting.

Independent variables

For the collection of sociodemographic data, information on age, gender, educational level and employment status was selected. Variables relating to the characteristics of caregiving were also included, such as the number of hours and years spent caring, the level of dependency of the person being cared for, whether the caregiver works shifts at the hospital, the length of hospital stay and the distance between the caregiver’s home and the hospital.

The burden perceived by caregivers was assessed using the short form of the Zarit Burden Interview. This instrument consists of seven items that assess the impact of caregiving on the caregiver using a five-point Likert scale, with response options ranging from 1 (never) to 5 (almost always). The total score is obtained by summing the responses to all items, such that higher scores indicate a greater level of burden. In accordance with the established criteria for interpretation, scores ≤16 were considered indicative of no burden, while higher scores reflected the presence of caregiver burden. The Zarit Brief Scale has demonstrated its validity and reliability in caregivers with different conditions [21]. In the Spanish population, the scale has shown adequate internal consistency, with a Cronbach’s alpha coefficient of 0.84 [22]. In the present sample, the internal consistency of the 7-item abbreviated version, assessed using Cronbach’s alpha, was 0.701. The factor structure of the abbreviated version of the Zarit scale was examined using exploratory factor analysis with principal axis factorization. The model fit was acceptable (KMO = 0.675), and Bartlett’s test of sphericity was significant (χ^2^(21) = 131.09; *p* < 0.001). The factor analysis supported a unidimensional structure.

Perceived social support was assessed using the Duke-UNC-11 Functional Social Support Questionnaire. This instrument comprises 11 items that explore different dimensions of perceived social support using a five-point Likert scale, ranging from 1 (“much less than I would like”) to 5 (“as much as I would like”). The total score ranges from 11 to 55 points, such that higher scores reflect a greater perception of social support. In accordance with the Spanish validation of the instrument, the 15th percentile was used as the cut-off point, with scores below 32 indicating a low level of perceived social support, while scores equal to or above this value reflect an adequate level of support. The Spanish version of the Duke-UNC-11 has demonstrated excellent psychometric properties, with high internal consistency (Cronbach’s α = 0.90). The questionnaire has been shown to be reliable and valid among caregivers in Spain [23]. Furthermore, the intraclass correlation coefficients obtained for the various items were greater than 0.50 in the Spanish adult population, which supports the reliability of the instrument [24]. The internal consistency of the Duke-UNC-11 questionnaire in the study sample, assessed using Cronbach’s alpha, was 0.696. Exploratory factor analysis revealed a two-factor solution. The data fit was acceptable (KMO = 0.611), and Bartlett’s sphericity test was significant (χ^2^(55) = 112.494; *p* < 0.001). Family functioning was assessed using the Family APGAR questionnaire, a widely used tool for evaluating individuals’ perceptions of how their family environment functions and their level of satisfaction with family relationships. The scale consists of five items using a three-point Likert-type response format, scored from 0 (“almost never”) to 2 (“almost always”). The total score allows family functioning to be classified into three levels: adequate functioning (7–10 points), mild family dysfunction (4–6 points) and severe family dysfunction (0–3 points). The version used has demonstrated adequate psychometric properties, with high internal consistency (Cronbach’s α = 0.84) [25]. Furthermore, its structural validity has been supported by satisfactory fit indices (χ^2^ = 7.90; *p* = 0.162; RMSEA = 0.04; TLI = 0.99; CFI = 0.99) in an adult population [26]. The internal consistency obtained in the study sample, assessed using Cronbach’s alpha, was 0.707. The exploratory factor analysis revealed a unifactorial structure. The data fit was good (KMO = 0.754), and Bartlett’s sphericity test was significant (χ^2^(10) = 75.807; *p* < 0.001).

Mental health status was assessed using the 12-item Goldberg General Health Questionnaire (GHQ-12), a screening tool designed to assess psychological distress in the general and clinical populations. The questionnaire consists of 12 items that explore the recent presence of emotional symptoms and behavioral disturbances. The items are answered using a four-point Likert scale (0–1–2–3), which can be converted into a dichotomous score (0–0–1–1), known as the GHQ score. The total score is obtained by adding up the items, with a possible range of 0 to 12 points in the case of a dichotomous score, such that higher scores indicate poorer mental health. Following the standard scoring method, a cut-off point of 12 points was established, considered indicative of the possible presence of clinically relevant psychological distress [27]. The version used corresponds to the Spanish adaptation of the GHQ-12, which has demonstrated adequate psychometric properties, with high internal consistency in the general population (Cronbach’s α = 0.86), as well as adequate indicators of construct validity and reliability across different population contexts [28,29]. This instrument has been extensively validated and is considered a reliable tool for screening mental health problems in epidemiological and clinical studies. The GHQ-12 showed an internal consistency in the study sample, assessed using Cronbach’s alpha, of 0.712.

### 2.5. Statistical Analysis

The data collected were coded and entered into a database designed specifically for the study. Continuous variables were described using means and standard deviations, while categorical variables were expressed as absolute frequencies and percentages. The distribution of quantitative variables was assessed using the Kolmogorov–Smirnov test. As the assumption of normality was not met, comparisons between caregivers who reported a need for support and those who did not were made using the Mann–Whitney U test for continuous variables and the χ^2^ test for categorical variables. Pearson’s chi-square test was used when the assumptions regarding expected cell frequencies were met. When these assumptions were not met because of small expected frequencies, Fisher’s exact test was used. Fisher’s exact test was applied to the relationship with the patient, educational level, distance from home to hospital, and dependency level of the care recipient.

A binary logistic regression was carried out to identify the factors associated with the perception of the need for care (0 = no; 1 = yes). Sample size adequacy for the regression analysis was assessed using the Events Per Variable (EPV) criterion [30]. Of the 94 caregivers included, 63 reported needing support (prevalence of 67.0%), and 31 did not; the less frequent event (n = 31) constitutes the limiting factor for estimating a stable model. Applying the criterion EPV ≥ 10, the maximum number of predictors that the available sample can support in a stable manner is approximately three (31/10 ≈ 3.1). The model was limited to three independent variables to avoid overfitting. Candidate predictor variables were preselected using bivariate analysis, considering as candidates those with *p* < 0.25, a recommended threshold as opposed to the traditional *p* < 0.05, which may exclude clinically relevant variables [31]. Consequently, the family APGAR score, the GHQ-12 score, and the length of hospital stay were included in the model as continuous variables.

The overall fit of the model was assessed using the −2 log-likelihood statistic and the Cox, Snell and Nagelkerke coefficients of determination. Calibration was examined using the Hosmer–Lemeshow test. The model’s discriminatory power was assessed using the area under the ROC curve (AUC). Multicollinearity among the independent variables was analyzed using the tolerance indices and the variance inflation factor (VIF), with VIF values < 5 and tolerance > 0.20 considered indicative of the absence of multicollinearity.

The results were expressed as β coefficients, odds ratios (OR), 95% confidence intervals (95% CI) and *p*-values. A sensitivity analysis was carried out by comparing the main model with an alternative model that excluded the GHQ-12. Statistical significance was set at a two-sided *p*-value ≤ 0.05. All analyses were performed using the Statistical Package for the Social Sciences (SPSS, version 25.0; IBM Corp., Armonk, NY, USA).

## 3. Results

Table 1 presents the sociodemographic characteristics of the participants and the variables related to the caregiving process during hospitalization, stratified according to the need for support expressed by the caregivers.

Comparative analysis between the two groups revealed statistically significant differences in the distance between the caregiver’s home and the hospital where the family member was admitted (*p* = 0.041), as well as in the length of hospitalization (*p* = 0.001). No significant differences were observed in the other variables analyzed.

Table 2 presents the results relating to caregiver burden and social support variables, as well as a comparison of these based on the need for support with caregiving tasks. The between-group analysis showed that caregivers who reported needing support had significantly higher levels of burden compared with those who did not require support (*p* = 0.034). Furthermore, statistically significant differences were observed in emotional well-being (*p* = 0.010), perceived social support (*p* = 0.014) and family functioning (*p* < 0.001), indicating a poorer psychosocial profile in the group requiring support.

The binary logistic regression analysis (Table 3) showed that family functioning and the length of hospital stay were significant predictors of the caregiver’s perceived need for support. Specifically, for every one-point increase in the Family APGAR score, the odds of perceiving a need for support decreased by approximately 25.1% (OR = 0.749; 95% CI: 0.602–0.932; *p* = 0.009), indicating a protective effect of better family functioning. Conversely, each additional day of hospitalization was associated with a 52.3 per cent increase in the odds of perceiving a need for support (OR = 1.523; 95% CI: 1.139–2.038; *p* = 0.005). The GHQ-12 score did not show a statistically significant association with the outcome (OR = 1.045; 95% CI: 0.959–1.138; *p* = 0.315).

The binary logistic regression model showed an adequate fit to the data (Table 4), with a −2 log-likelihood of 92.750, a Cox and Snell R^2^ of 0.245 and a Nagelkerke R^2^ of 0.341. The Hosmer–Lemeshow goodness-of-fit test did not indicate any lack of calibration in the model (χ^2^ = 10.921; df = 8; *p* = 0.206). Furthermore, the model demonstrated good discriminatory power, with an area under the ROC curve (AUC) of 0.817 (95% CI: 0.718–0.917; *p* < 0.001). No missing data were identified; therefore, all 94 participants were included in the analysis (63 events and 31 non-events). The assessment of multicollinearity revealed no issues amongst the independent variables, with tolerance values ranging from 0.918 to 0.985 and VIF values ranging from 1.015 to 1.089.

A sensitivity analysis was carried out excluding the GHQ-12 variable (Table 5), as it did not show a statistically significant association in the main model. The results were similar to those of the main model: the family APGAR score and length of stay remained significantly associated with the caregiver’s need for support, with comparable OR estimates and confidence intervals. Furthermore, the Nagelkerke R^2^ (0.341 vs. 0.329) and the Hosmer–Lemeshow test (*p* = 0.206 vs. *p* = 0.506) showed similar patterns in both models.

## 4. Discussion

This study analyzed the factors associated with the need for support among informal caregivers of hospitalized patients using a multivariate logistic regression model. The results show that this need is independently associated with two key dimensions: family functioning (Family APGAR) and length of hospital stay.

In this regard, better family functioning was independently associated with a lower likelihood of needing support. This finding suggests that a more structured and functional family environment may be linked to a greater ability to adapt to the demands of caregiving and to a lower perceived need for support on the part of the caregiver.

These results are consistent with recent studies that have described associations between lower family functioning and higher levels of burden, fatigue, and deterioration in the caregiver’s well-being [32,33]. The available evidence suggests that families with better communication, greater organizational capacity, and a more balanced distribution of caregiving tasks cope more effectively with the demands arising from the caregiving process. This allows care to be maintained over extended periods with a reduced need to rely on external support [33]. From a systemic perspective, caregiving cannot be understood solely as an individual responsibility but rather as a relational phenomenon that unfolds within a specific family structure. Family functioning acts as a buffer against the stress of caregiving, fostering shared coping strategies and reducing the isolation of the primary caregiver.

The recent literature broadly supports this association. A systematic review identified that intensity of care, the time spent providing care, the patient’s level of dependence, and insufficient family resources constitute some of the main determinants of the caregiver’s needs for support and perceived burden [34]. Similarly, various studies have shown that an increase in burden is associated with a decrease in quality of life, a greater perception of exhaustion, and a greater need for external support [17,32,35].

The absence or limitation of family support networks and effective support mechanisms can reduce the caregiver’s coping capacity [36]. Furthermore, it has been found that this type of caregiver is more likely to experience a negative impact on their health than those who do have support networks; therefore, this situation highlights the need to provide support to caregivers [37].

The present study extends this evidence to the context of prolonged hospitalization, where caregivers not only face caregiving tasks but also family reorganization, clinical uncertainty, and continuous adaptation to changes in the patient’s condition. In this context, the need for support could be interpreted as a manifestation of the caregiver’s functional limit in maintaining a balance between the demands of caregiving and their own personal resources.

The length of the hospital stay was also independently associated with the caregiver’s reported need for support. Specifically, longer hospital stays were associated with a higher probability of needing support. This association may reflect the fact that longer caregiving processes are often accompanied by greater organizational and emotional demands on caregivers.

Although this factor has been studied less specifically in hospital caregivers, the available evidence suggests that prolonged hospitalization progressively increases the organizational, emotional, and caregiving demands on the caregiver. Studies conducted on caregivers of hospitalized patients have described higher levels of burden, lower preparedness for care, and increased support needs as the complexity and duration of the care process increase [10,28,38]. In this context, the ongoing involvement of caregivers positions them as key figures within the care process; they may be exposed to greater emotional and organizational demands, uncertainty regarding clinical progress and discharge, as well as a need for information, preparation and professional support [28,38].

In line with these findings, a longitudinal study carried out in hospital medical wards showed that the involvement of informal caregivers was greater amongst patients at risk of prolonged hospitalization, anticipated difficulties with discharge, and a higher incidence of adverse events. These results highlight that families play a significant caregiving role during hospitalization, particularly in more complex cases, through activities such as accompanying the patient, monitoring their condition and supporting their needs [39].

One possible explanation is that prolonged hospitalization involves a progressive accumulation of care demands, travel, work interruptions, changes in family routines, and greater clinical uncertainty. From this perspective, the duration of hospitalization could act as an indirect marker of care complexity and serve as a criterion for prioritizing nursing interventions aimed at the caregiving environment.

Similarly, a study of caregivers of hospitalized older adults identified ethnocultural differences in the amount of time spent with patients during the day, overnight stays, and oversight of health care. These findings show that family involvement during hospitalization can be intense and take different forms depending on cultural norms. Taking these differences into account is essential for fostering respectful and effective coordination between family caregivers and healthcare professionals [40].

In other areas of healthcare, it has also been observed that cultural and religious beliefs can influence the public’s attitudes towards certain health-related decisions. Various studies show that these decisions do not depend solely on clinical information, but are also influenced by emotional, cultural and ethical factors that healthcare professionals must take into account when supporting patients and their families [41,42].

In our study, the caregiver’s mental health, as assessed using the GHQ-12, was not independently associated with the need for support following adjusted analysis. This finding is consistent with the model of the caregiver’s stress process proposed by Pearlin et al. [20] according to which emotional well-being does not depend on a single factor, but rather results from the interaction between care-related stressors, available personal and social resources, and coping mechanisms. In line with this conceptual framework, recent evidence shows that greater perceived social support is associated with lower levels of depressive and anxiety symptoms among informal caregivers, while subjective burden and depressive symptoms, although closely related, represent distinct constructs within the caregiving experience [19]. Furthermore, perceived social support and support received constitute different, though complementary, dimensions of support for caregivers. Taken together, these findings suggest that the need for support and emotional well-being are closely related but conceptually different dimensions, whose association may cease to be independent when other factors involved in the caregiving experience are considered simultaneously.

In this context, caregiver support currently represents a significant challenge in healthcare, as it requires the early identification of situations of vulnerability that may contribute to an increase in the need for support. During hospitalization, caregivers also face new emotional, organizational and relational demands arising from uncertainty about the patient’s prognosis, communication with healthcare professionals, disruption to family routines and the need to be physically present at the hospital. In this regard, the results of this study suggest that this need does not depend solely on the caregiver’s individual condition but is primarily influenced by family functioning and the characteristics of the care process, particularly the length of the hospital stay. A longer hospital stay appears to increase the demands on care, while better family functioning makes it easier to manage the situation and share responsibilities. Therefore, the incorporation of a systematic assessment of the caregiver and the family environment during hospitalization could help prevent situations of family breakdown, improve the caregiver’s well-being, and promote safer, more comprehensive, and sustainable care for those being cared for.

### 4.1. Study Limitations

Regarding the limitations of this study, the small sample size influenced the specification of the final logistic regression model, which was restricted to three predictors, in accordance with the recommended ratio of EPV, to improve model stability and reduce the risk of overfitting. However, this restriction may have affected the precision of the estimates, as reflected in the width of the confidence intervals. Therefore, the results should be interpreted with caution and validated in future studies with larger samples.

On the other hand, the cross-sectional design prevents the establishment of the temporal direction or causality of the observed associations. Furthermore, the assessment of overload, psychological distress, social support, family functioning and the need for support using self-report questionnaires administered at a single point in time may have introduced common-method bias and influenced the magnitude of the associations found. Responses may also have been affected by social desirability, which could lead to an underestimation or overestimation of experiences, particularly regarding issues related to family functioning and the ability to provide care. Furthermore, the absence of longitudinal data prevents an examination of changes in caregiver burden, perceived social support, psychological distress and family functioning over time, which hinders a comprehensive understanding of the dynamic nature of these factors. In addition, the under-representation of participants in certain categories of distance between home and hospital may have reduced the accuracy of the corresponding estimates. The study was conducted within a specific regional context, whose socio-demographic, organizational and healthcare characteristics may differ from those in other settings. While this does not compromise the validity of the results for the study population, it should be borne in mind when extrapolating them to other geographical areas and healthcare systems.

Future longitudinal and multicenter studies should examine the progression of caregiver burden and their support needs from hospital admission through to discharge and during the return home. They would also enable an analysis of the influence of family, organizational and care-related factors on the caregiving experience, as well as testing the reproducibility of the findings across different healthcare and geographical contexts.

### 4.2. Study Strengths

One of the main strengths of this study is its multidimensional approach to the phenomenon of informal care, as it simultaneously incorporates variables related to caregiver burden, psychological distress, perceived social support, family functioning, and characteristics of the care process. This approach provided a broader view of the factors associated with the caregiver’s need for support during hospitalization.

In this sense, this study provides evidence in a context that remains largely unexplored in the literature: the informal caregiver of the long-term hospitalized patient. From a clinical and nursing perspective, the results obtained may contribute to improving the early identification of vulnerable caregivers and guide the design of interventions aimed at preventing caregiver burden and promoting the well-being of the caregiving environment.

### 4.3. Implications for Nursing

From a clinical and nursing perspective, the findings may help to improve the early identification of vulnerable caregivers and guide the design of interventions aimed at promoting well-being in the care setting.

The findings could inform a nursing assessment approach for informal caregivers during hospitalization. The nurse in charge could identify the primary caregiver and carry out an initial screening for perceived burden, family functioning, psychological distress and the need for support during the early stages of admission. This assessment could be updated periodically during prolonged stays, in the event of significant clinical or family changes, and prior to discharge.

Where risk situations are identified, the nurse could carry out a more detailed assessment and coordinate the response with the multidisciplinary team. Educational and care needs could initially be addressed by the nursing team, while social, emotional or continuity-of-care issues may require the involvement of healthcare social work, clinical psychology, the case management nurse, the medical team or primary care services. Possible interventions would include health education, care training, advance discharge planning, family meetings to allocate responsibilities, encouraging rest periods, preparing in advance for the transition home, and referral to social or community resources. Given the observational design of the study, this proposal should be regarded as indicative, and its applicability and effectiveness will need to be evaluated in longitudinal studies and through intervention trials.

## 5. Conclusions

The need for support among informal caregivers during a prolonged hospital stay was primarily associated with family functioning and the length of the hospital stay. Specifically, longer hospital stays were associated with a greater need for support, while better family functioning was associated with a lower need for support.

Overall, these findings highlight the need to involve informal caregivers in the clinical care of patients requiring prolonged hospitalization, through a holistic approach that includes not only the patient but also the caregiver and their family environment throughout the entire care process. Systematic assessment of family functioning and support needs could enable healthcare teams to identify vulnerable caregivers at an earlier stage and implement timely psychosocial, educational, and organizational interventions aimed at preventing family breakdown, improving the caregiver’s well-being, and promoting safer and more sustainable care. These findings should be interpreted with caution due to the study’s cross-sectional design, the relatively small sample size, the geographic scope of the sample, and the self-reported nature of the primary outcome measure. Future longitudinal studies with larger and more diverse samples are needed to confirm the observed associations.

## Figures and Tables

**Table 1:** Sociodemographic characteristics and care-related variables according to caregiver’s need for support.

	Total			Needs Support			Does Not Need Support			χ^2^/Fisher (*p*)
	*n*	%		*n*	%		*n*	%		
**Gender**										0.178 (0.673)
Male	16	17		10	15.9		6	19.4		
Female	78	83		53	84.1		25	80.6		
**Relationship**										1585 (0.654)
Son/Daughter	69	73.4		45	71.4		24	77.4		
Partner	13	13.8		8	12.7		5	16.1		
Father/mother	2	2.1		2	3.2		0	0		
Other	10	10.6		8	12.7		2	6.5		
**Level of education**										5.729 (0.457)
Unable to read or write	5	5.3		4	6.3		1	3.2		
Primary education	43	45.7		29	46.0		14	45.2		
Secondary education	9	9.6		7	11.1		2	6.5		
Upper secondary education	15	16		12	19.0		3	9.7		
Intermediate vocational education and training (VET)	11	11.7		6	9.5		5	16.1		
Higher vocational education and training (VET)	6	6.4		2	3.2		4	12.9		
University education	5	5.3		3	4.8		2	6.5		
**Employment status**										4.591 (0.101)
Unemployed	24	25.5		19	30.2		5	16.1		
Employed	52	55.3		30	47.6		22	71.0		
Retired	18	19.1		14	22.2		4	12.9		
**Distance from home to hospital**										7.237 (0.063)
<5 km	33	35.1		20	31.7		13	41.9		
5–20 km	25	26.6		14	22.2		11	35.5		
20–50 km	21	22.3		15	23.8		6	19.4		
>50 km	15	16		14	22.2		1	3.2		
**Level of dependency of the cared-for person**										0.925 (0.553)
Independent or mild dependency	6	6.4		5	7.9		1	3.2		
Moderate dependency	27	28.7		19	30.2		8	25.8		
Severe dependency	61	64.9		39	61.9		22	71.0		
**Hospital shift work**										1.021 (0.312)
Yes	60	63.8		38	60.3		22	71.0		
No	34	36.2		25	39.7		9	29.0		
	***n***	**Median**	**IQR**	***n***	**Median**	**IQR**	***n***	**Median**	**IQR**	**U (*p*)**
**Age (years)**	94	56	19.25	63	57	18	31	51	20	882.5 (0.449)
**Years of caregiving**	94	3	1	63	3	2	31	2	1	922 (0.648)
**Hours of caregiving**	94	9	10	63	8	9	31	10	16	851 (0.306)
**Length of hospital stay**	94	11	3	63	12	1	31	10	2	400 (0.001)

**Table 2:** Variables relating to stress, emotional well-being and social and family support, broken down by caregivers who reported needing support and those who did not.

	Total			Needs Support			Does Not Need Support			
	*n*N	Median	IQR	*n*	Median	IQR	*n*	Median	IQR	U (*p*)
Zarit Burden Interview	94	18	6	63	19	2	31	13	1	502 (0.034)
Goldberg General Health Questionnaire (GHQ-12)	94	11	4	63	11	1	31	8	1	853.5 (0.010)
Duke-UNC-11 Functional Social Support Questionnaire	94	37	4	63	37	5	31	40	3	484 (0.014)
Family Apgar	94	7	3	63	6	2	31	8	2	454.5 (0.000)

U: *p*-value from U Mann–Whitney.

**Table 3:** Association between the need for support among caregivers of family members with prolonged hospital stays and mental health status, family functioning and length of stay.

	β	SE	Wald	*p*-Value	OR	95% CI
Goldberg General Health Questionnaire (GHQ-12)	0.044	0.044	1.010	0.315	1.045	0.959–1.138
Family Apgar	−0.289	0.111	6.734	0.009	0.749	0.602–0.932
Length of stay	0.421	0.148	8.040	0.005	1.523	1.139–2.038

SE, standard error; OR, odds ratio; CI, confidence interval.

**Table 4:** Performance and goodness-of-fit of the binary logistic regression model.

Statistic	Value
−2 Log likelihood	92.750
Cox & Snell R^2^	0.245
Nagelkerke R^2^	0.341
Hosmer–Lemeshow χ^2^ (df = 8)	10.921
Hosmer–Lemeshow *p*-value	0.206
Area under the ROC curve (AUC)	0.817
95% CI for AUC	0.718–0.917
AUC *p*-value	<0.001
Events	63
Non-events	31
Missing data	0
Tolerance	0.918–0.985
Variance Inflation Factor (VIF)	1.015–1.089

**Table 5:** Sensitivity analysis.

Variable	Main Model OR (95% CI)	*p*-Value	Sensitivity Model OR (95% CI)	*p*-Value
Family APGAR	0.749 (0.602–0.932)	0.009	0.730 (0.592–0.901)	0.003
Length of stay	1.523 (1.139–2.038)	0.005	1.587 (1.190–2.116)	0.002
GHQ-12	1.045 (0.959–1.138)	0.315	-	-
Nagelkerke R^2^	0.341		0.329	
Hosmer–Lemeshow (*p*)	0.206		0.506	

## Data Availability

The data are included within the article.

## Abbreviations

The following abbreviations are used in this manuscript:

GHQ-12	Goldberg General Health Questionnaire
Duke-UNC-11	Duke-UNC-11 Functional Social Support Questionnaire
